# The Relations of Operative Gynecology to Insanity

**Published:** 1893-07

**Authors:** A. H. McFarland

**Affiliations:** Jacksonville, Ill.


### THE RELATIONS OF OPERATIVE GYNECOLOGY TO INSANITY.*

BY A. H. McFARLAND, M. D.,
JACKSONVILLE, ILL.

After stating that the ideal aim of the progressive alienist should be the solution of the great problem of the physical foundation of insanity, the essayist quoted from Dr. Maudsley: "We recognize how entirely the integrity of the mental functions depends upon the bodily organization, and as physicians we cannot afford to lose sight of the physical aspects of mental states, if we would truly comprehend the nature of mental disease. We recognize the existence of an intelligent mental force, linked in harmonious association and essential relations with other forces, but leading and constraining them and led and constrained by them in its manifestations." Dr. Spitzka has stated that, "disordered states of the pelvic organs have been supposed to play an important part in the causation of insanity. It is known, however, that the grossest lesions of the uterine region are not usually complicated by such mental disturbance as justifies calling it alienation. Those pretty cases, in which a delusional insanity is instantaneously cured by restoring a retroflected or retroverted uterus to a normal position, do not seem to occur nowadays, and the gynecological epoch of psychiatry

*Abstract of a paper read before the American Medical Association, Milwaukee.

seems to have passed by, taking its adieu with the sacrifice at Blackwell's Island Asylum of Mary Ann Mullen, a sufferer from unrecognized katatonia on the altar of oöphorectomy. It would have been as reasonable to extirpate the bedsore of a sufferer from paretic dementia, or cut off the hematomatous ear of a terminal dement, with the hope of curing his insanity thereby."

Dr. Richard Dewey says: "The continuous and very considerable increase of insanity throughout our country, and the somewhat discouraging results of treatment, have led to dissatisfaction and to seeking after new ideas and methods and this tendency has been further stimulated of late by the great advance in neurology, neuropathology pyschopathology and operative cerebral surgery. This general recognition of the defects of present methods, and search for newer and better ones has, like all things human, its good and evil side. It is undoubtedly a good thing in itself, but there has been a disposition on the part of more recent converts to an interest in nervous and cerebral diseases, to discard all past and present ideas as antiquated and insufficient, and to form extravagant expectations or results from newer applications, while at the same time, those already occupying the field have been perhaps too little aware of the possibilities brought within reach by the discoveries of recent years. These facts are illustrated by the abortive movement inaugurated a short time ago in London to establish an asylum for the insane, to be solely managed by a visiting staff of specialists in various branches, with not a man among them connected with any institution for the insane, or possessing a practical knowledge of the care and management of the insane, and there are numerous illustrations of the same kind in our own country. The men engaged in private practice in the specialties of brain and nervous diseases on the one hand, and the men in charge of institutions for the insane on the other, have, in the past, affiliated to only the slightest degree, and have misunderstood each other. And furthermore, the former have furnished most striking illustrations of their practical ignorance of insanity and its management, marked by calamitous results among their patients, while the latter have been found wanting in the scientific spirit and have too often been absorbed in purely administrative matters, while the rich opportunities for pathological study and for original research have failed to be improved.

*L'uterus c'est la femme* is a proverb which has received a new development in these days; for if, by courtesy rather than by conviction, women be granted the possession of a few subsidiary organs, these, at best, have no prerogative nor any order of their own. The uterus has its maladies of local causation, of nervous and mixed causation as other organs have; but to assume that all uterine neuroses or even all general neuroses in women are due to changes in the uterus, is as dull as to suppose that the stomach can never be the seat of pain except it be the seat of some local affection. While pelvic and abdominal diseases of women are more frequently and intelligently recognized by the profession now than formerly, it may be affirmed that more women, ten to one, have been consigned to hospitals for the insane (victims of needless or unskillful surgical operations) than have ever been restored to reason by the most commendable and skillful abdominal surgery. The essayist did not deny that insane women are capable of suffering from local disorders, and that surgery offers no hope of amelioration to these unfortunate women. In some cases anesthesia may exist, masking conditions so that no suspicion of their nature is entertained; in other cases hyperesthesia makes the most trifling ailments appear as serious, while no doubt there is a large number of mentally sick women suffer, and are conscious that they suffer, from some local disorder, manifested to them in the form of pain or irritation.

Hysterical mania is not an uncommon complication of the diseases of woman. Two months ago such a case came under my observation with the following history: Mrs. H., age thirty, admitted to hospital February 7, 1893, married four years, three children, no history of insanity in family. Birth of first child produced laceration of third degree.

Perineorraphy performed two months after delivery, leaving the patient in a weakened and nervous condition. Whitehead's operation, to which she submitted under protest, was then performed. Within two months she became so insane that a verdict was easily obtained. My diagnosis could be easily made as a case of hysterical mania, aggravated by local treatment of a year, and two unsuccessful operations.

Dr. J. M. Baldy read a paper before the American Gynecological Society on "Insanity following Laparotomy," in which he gives statistics from the insane asylums of Pennsylvania, in regard to number of patients received after laparotomy. From eighteen institutions, he received reports of fifteen cases, and reported one of his own for retocele, after which the patient developed melancholia and another, having chronic confusional insanity after oöphorectomy.

Four possible dangers should be considered: 1. Previous insane taint. 2. The anæsthetic. 3. Fear of the operation. 4. Sepsis.

The essayist did not believe one factor sufficient to account for a serious result.

The chief medical error of the present day is the mistaking of brain disease for pelvic disease, and the prayer of the neurologist in behalf of the neurasthenic woman is for rest for her whole organism from needless irritation and unnecessary gynecological operative disturbances. This does not include a protest against the treatment of the real consequences of gynecian disease nor of those uterine disorders which are the result of neuropathic disorders, the hyperemias, hyperplasias, morbid growths, grave traumatisms and displacements. These are surely numerous enough to keep gynecologists employed.

The day of gynecological errors is waning; a light shines from behind the cloud. It is the ray of the neurological sun, which, through clouds, dawns for woman's emancipation from the misery of medical error, and a true neurological gynecology is born into the family of clinical and therapeutic science.

CONCLUSIONS.

1. Gynecological operations are more likely than any other surgical procedures to disturb the mind.

2. Hereditary antecedents of the patient should always be determined.

3. In insane patients, operations should be performed only when the physical condition endangers or renders life insupportable.

4. Patients, preceding the operation, should be in a calm frame of mind; hence, moral treatment of the patient previous to operating is the best prophylaxis.

5. Inherited and acquired insane constitution is the fundamental factor in most cases of insanity. This conclusion does not, however, justify us in ignoring physical diseases, immediately preceding or associated with insanity.

6. Healthy genital organs do not give rise to reflex symptoms, consquently caution should be exercised in operating for the relief of insanity.

7. Operations may be satisfactory in properly selected cases.

